# Successful Treatment of Refractory Thrombotic Thrombocytopenic Purpura (TTP) With Caplacizumab: A Case Report

**DOI:** 10.7759/cureus.42423

**Published:** 2023-07-25

**Authors:** Debolina Pramanik, Divyashish Bhardwaj, Vikash K Karmani, Girindra G Raval, Abdullah Kutlar

**Affiliations:** 1 Medicine, Medical College Kolkata, Kolkata, IND; 2 Medicine, Jawaharlal Nehru Medical College, Aligarh, IND; 3 Internal Medicine, Jinnah Sindh Medical University, Karachi, PAK; 4 Medicine: Hematology and Oncology, Medical College of Georgia at Augusta University, Augusta, USA

**Keywords:** caplacizumab, rituximab, plasmic score, systemic lupus erythematosus, refractory thrombotic thrombocytopenic purpura

## Abstract

We report a female patient who presented with generalized weakness, episodes of altered mental status and slurred speech, and a history of systemic lupus erythematosus. Initial investigations showed profound thrombocytopenia and schistocytosis on peripheral blood smear. PLASMIC score was promptly calculated, and plasma exchange with steroids was initiated based on the initial high PLASMIC score. Bone marrow examination showed hypocellular marrow without any other obvious abnormalities. The patient’s platelet counts initially improved but had a quick decline, on which, rituximab and subsequently caplacizumab were introduced. The patient was discharged after stabilization with plasma exchange (PLEX) therapy as needed on an outpatient basis.

## Introduction

Thrombotic thrombocytopenic purpura (TTP), a subtype of thrombotic microangiopathy, is a rare hematologic condition that leads to severe thrombocytopenia and occlusive microangiopathy with potential sequelae including fever, neurological abnormalities, renal insufficiency, thrombocytopenia, end organ damage, and death if untreated. The disease is caused by decreased activity of ADAMTS13 (a disintegrin and metalloproteinase with a thrombospondin type 1 motif, member 13), which is a von-Willebrand Factor cleaving protease, and may be either acquired (due to the presence of an inhibitor or antibody to ADAMTS13) or congenital (due to decreased production of ADAMTS13 from a genetic mutation) [1].

Thrombotic thrombocytopenic purpura has an incidence of 3-11 cases per million and a prevalence of 10 cases per million and is most common in the fifth decade of life in the USA. If untreated, the mortality is 90% which significantly declines to 10%-15% if treated [1].

Refractory TTP is defined as failure of platelet response to plasma exchange (PLEX) therapy and immunosuppressants after 4-7 days of treatment, or worsening of symptoms after treatment with PLEX and immunosuppressants for 7 days, and comprises 20% of all cases [1].

Patients with systemic lupus erythematosus (SLE) have a higher risk of developing TTP, with an incidence of 0.5%-2%. They also have a higher mortality rate (34%-62.5%) as compared to idiopathic TTP (20%), even after treatment [2].

Thrombotic thrombocytopenic purpura often requires a presumptive diagnosis based on microangiopathic hemolytic anemia (MAHA) and thrombocytopenia, as it requires immediate life-saving treatment such as PLEX and immunosuppressive therapy [3].

Diagnosis is confirmed when ADAMTS13 activity is found to be severely low (<10%). Since the test has a prolonged turnaround time, it leads to a delay in confirming the diagnosis. Therefore, a good indicator for stratifying the risk of TTP is the PLASMIC score [platelet count; combined hemolysis variable; absence of active cancer; absence of stem-cell or solid-organ transplant; mean corpuscular volume (MCV); international normalized ratio (INR); creatinine]. Each point is given a score of 1 and patients are stratified into low (0-4), intermediate (5), and high (6-7) risk of TTP [3].

Other therapies with varying levels of success include intravenous immunoglobulin (IVIG), cyclosporine, vincristine, and bortezomib. A splenectomy may also be tried as a last resort [1].

Here we describe a case of a patient with refractory TTP.

## Case presentation

A 56-year-old African American female patient presented with generalized weakness, episodes of altered mental status, and slurred speech for the last 6 days. She had a past medical history of hypothyroidism, SLE, hypertension, and dyslipidemia. Her home medications include hydroxychloroquine, prednisone, celecoxib, thyroxine, vitamin D3, and hydrochlorothiazide.

Her surgical, family, and social history were unremarkable, without any known medication allergies.

On physical examination, she was stable without any acute distress. Vital signs including orthostatics were within normal limits. She was alert and oriented to time, place, and person (Glasgow Coma Scale Score - 15).

Her skin was normal for ethnicity without any visible rash on exposed skin, including malar rash. She did not have any pallor or yellowish discoloration of the skin. Cardiovascular examination showed normal S1 and S2, without any extra heart sounds. She had well-perfused extremities, without any peripheral edema. Her lungs were clear to auscultation bilaterally, without any wheezing or crackles. Gastrointestinal examination showed no hepatosplenomegaly with normal bowel sounds in all four quadrants. She had a normal range of joint movement without any swelling, tenderness, or deformities. A neurological examination did not reveal any abnormalities. There was no evidence of active oral, vaginal, or rectal bleeding.

Investigations

Initial investigations showed anemia with hemoglobin of 9.3 g/dL with profound thrombocytopenia of 11 x 103/mm^3^. Her peripheral blood smear showed marked polychromasia and mild schistocytosis, with moderate anisocytosis, moderate microcytosis, mild hypochromia, moderate poikilocytosis, and a high reticulocyte count of 7.0% with red cell distribution width (RDW) of 19.3%.

With TTP as a suspected cause, the PLASMIC score [3] was calculated as shown in Table 1:

**Table 1:** PLASMIC scoring system and patient's PLASMIC score calculation. INR, international normalized ratio; MCV, mean corpuscular volume

PLASMIC (platelet count; combined hemolysis variable; absence of active cancer; absence of stem-cell or solid-organ transplant; MCV; INR; creatinine) score		
Criteria	Patient’s value	Score
Platelet count <30 x 10^3^/L	Yes (11 x 10^3^/mm^3^)	1
Hemolysis [reticulocyte count >2.5%, haptoglobin undetectable, or indirect bilirubin >2.0 mg/dL (34.2 µmol/L)]	Yes (reticulocyte count >7%)	1
Active cancer	No	1
History of solid-organ or stem-cell transplant	No	1
MCV < 90 fL	Yes (78.1 fL)	1
INR < 1.5	Yes (1.1)	1
Creatinine < 2 mg/dL	Yes (0.94 mg/dL)	1
PLASMIC score		7
CONCLUSIONS: Risk group: High risk, Risk of severe ADAMTS13 deficiency (defined as ADAMTS13 activity level <15%): 72%		

**Figure 1:**
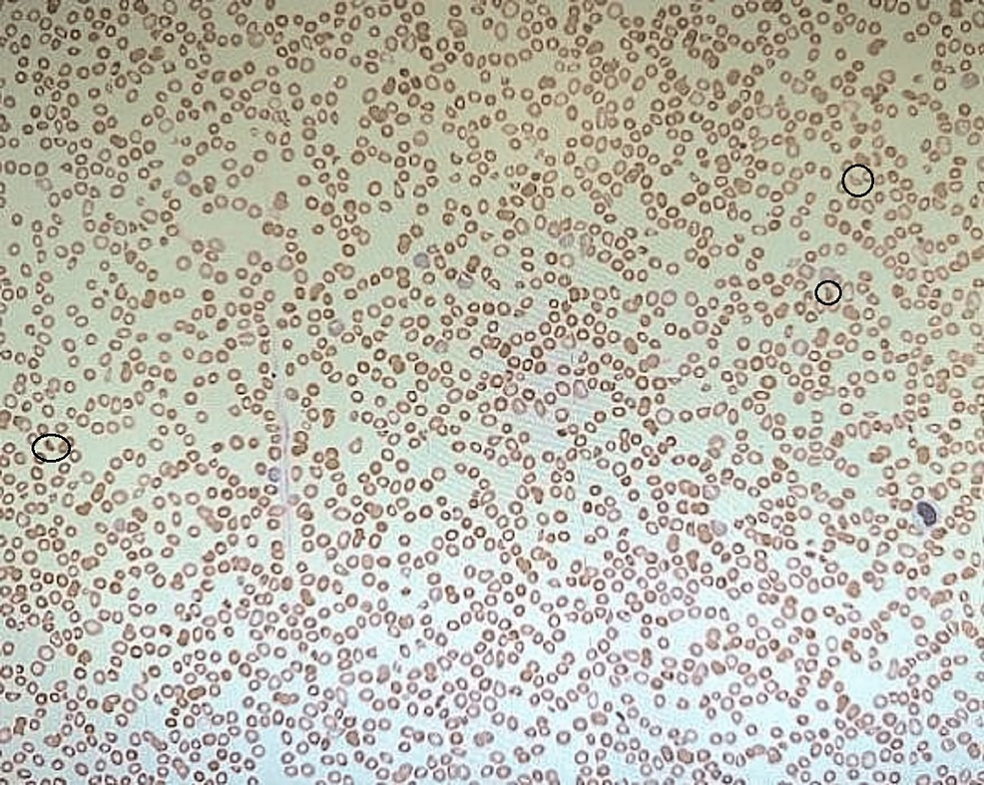
Peripheral blood smear of the patient on admission (the schistocytes are circled). Black circle: schistocyte

The peripheral blood smear of the patient is shown below in Figure 1:

Some of her pertinent test results are mentioned below as shown in Tables 2-3:

**Table 2:** Complete blood count of the patient. RDW, red cell distribution width; MCHC, mean corpuscular hemoglobin concentration; MCH, mean corpuscular hemoglobin; MCV, mean corpuscular volume; RBC, red blood cells; WBC, white blood cells

Complete blood count parameters	01/20/2023
WBC	4.7 x 10^3^/mm^3^
RBC	3.53 x 10^6^/mm^3 ^(low)
Hemoglobin	9.3 g/dL (low)
Hematocrit	27.6% (low)
MCV	78.1 fL (low)
MCH	26.5 pg (low)
MCHC	33.9 g/dL
RDW	19.3% (high)
Platelet count	11 x 10^3^/mm^3^ (critical)
Reticulocyte count	7.0% (high)

**Table 3:** Liver and kidney function tests of the patient. LDH, lactate dehydrogenase; eGFR, estimated glomerular filtration rate; BUN, blood urea nitrogen

Liver and kidney function parameters	01/20/2023
BUN	18 mg/dL
Creatinine	0.94 mg/dL
Total bilirubin	1.3 mg/dL (high)
Direct bilirubin	0.2 mg/dL
LDH	702 U/L (high)
eGFR	74 mL/min/1.73m^2^

Her thyroid function tests, vitamin B12, and folate studies were found to be within normal limits. Coombs' test was negative. As plasma exchange (PLEX) therapy had already begun, the SLE antibody profile including anti-dsDNA could not be done.

Differential diagnoses

The patient's initial presentation of episodes of altered mental status and slurred speech included differential diagnoses like cerebrovascular stroke, hypovolemic shock, hypoglycemia, and diuretic-induced electrolyte imbalances.

While considering TTP, the most common differential diagnoses of TTP-like diseases include hemolytic uremic syndrome (HUS)), disseminated intravascular coagulation (DIC), and malignant hypertension [4]. However, the patient's age and lack of any preceding infectious diseases; normal prothrombin time (PT) and INR; and normal chest X-ray, and liver function tests helped rule out these causes respectively.

A few other causes that may present with MAHA with thrombocytopenia include preeclampsia with severe features and hemolysis, elevated liver enzymes, low platelets (HELLP) syndrome; systemic infections like bacterial endocarditis, HIV, malaria, and babesia; rheumatic diseases such as SLE and antiphospholipid syndrome (APLS); and organ transplant complications [4].

Her urine pregnancy test was found to be negative, ruling out pregnancy-related issues. While SLE might be related to the patient's episode of TTP, the SLE-related features were under control with no acute exacerbations on her current medications [5].

Quinine is the most common cause of immune-mediated drug-induced thrombotic microangiopathy (DITMA). Gemcitabine, cyclosporine, mitomycin C, bevacizumab, ticlopidine, and clopidogrel are some of the other causes of drug-induced TMA [4, 6]. To avoid drug-related thrombocytopenia, her hydroxychloroquine and celecoxib were temporarily held on admission.

Other rarer causes include disorders of vitamin B12 metabolism or factors involved in hemostasis [4].

Treatment

After drawing blood for ADAMTS13 activity assay, PLEX therapy was ordered to begin immediately along with 100 mg of methylprednisolone based on the suspected TTP. Platelet transfusion and fresh frozen plasma transfusion were also ordered if needed, however, the patient did not receive any platelet transfusions during the course of her hospital admission. Her ADAMTS13 activity assay was found to be <1% and ADAMTS antibody titer was positive at 42 BU (Bethesda Units)/mL. These findings confirmed the diagnosis of acquired TTP. 

For Days 2-5, prednisone 80 mg/day and PLEX therapy were continued. The patient showed a remarkable response to therapy with a platelet count of 60 x 103/mm3 as on Day 5. While PLEX therapy was continued daily, prednisone was switched to methylprednisolone 1 g daily, with the platelet count of the patient rising to 123 x 103/mm3 as on Day 8.

However, a sudden drop in the platelet count to 74 x 103/mm3 occurred on Day 9. This clinical course of the patient led to the categorization of the disease as refractory TTP, and the decision to begin Rituximab once weekly was taken, beginning 29th January 2023 (Day 9). The platelet count began showing a slow rise to 83 x 103/mm3 as on Day 11.

**Figure 2:**
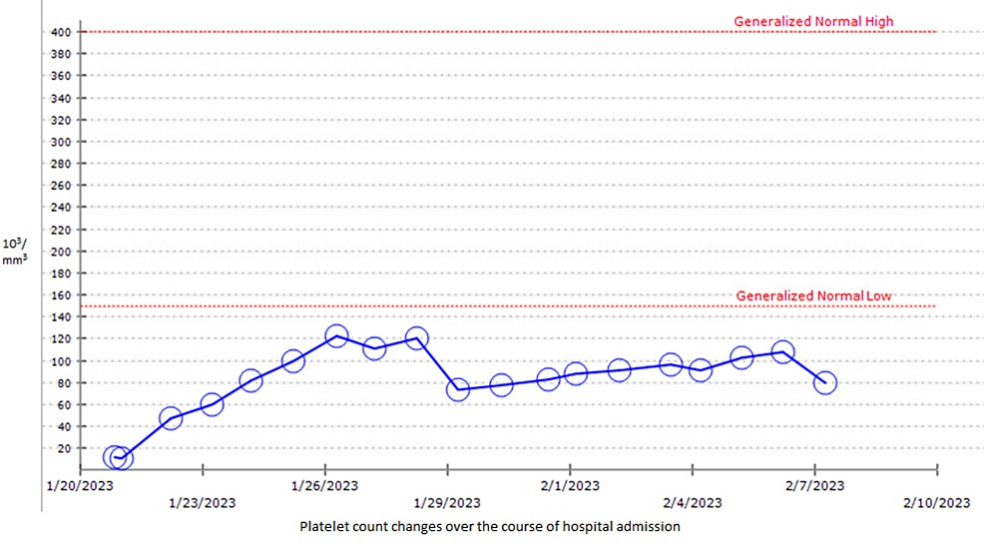
Platelet count of the patient over the course of hospital admission.

A graph of the patient’s platelet count over the course of her admission is shown below in Figure 2:

Bone marrow examination revealed hypocellular marrow (10%) with sparse maturing marrow elements and it was negative for acute leukemia, lymphoma, and atypical infiltrates without any cytogenetic abnormalities. The findings were suggestive of a reactive suppressive process. The differentials include - aplastic anemia, drug/toxin effect, infection, nutritional deficiency, and immune suppression among others.

Anti-vWF (von Willebrand factor) therapy caplacizumab 11 mg SQ (subcutaneous) four times daily was begun on 2nd February 2023 (Day 13) along with the continuation of her ongoing medications. Her ADAMTS13 level improved to 81% on 7th February 2023. 

As on Day 17, the patient was stable with vitals in the normal range, including her blood pressure, Hb level of 7.4 g/dL, and platelets increased to 108 x 103/mm3.

Since then, her platelet count has remained in the range of 80 x 103/mm3-110 x 103/mm3. PLEX therapy was held on Days 21 and 22 to assess the patient's platelet response, who remained stable without a drop in platelet count or any active bleeding. The patient was discharged on Day 23 with a plan to continue PLEX therapy if needed on an outpatient basis. The patient had received a total of three doses of Rituximab, and 6 days of treatment with caplacizumab during the course of her hospital stay. Also, the steroid dose taper and her home medications of SLE including hydroxychloroquine were begun before discharge. She also received another dose of Rituximab 1 week post-discharge. There was no relapse of TTP after caplacizumab withdrawal, and she has been in remission since.

## Discussion

While the classical presentation of TTP has been described with the Moschowitz clinical pentad as mentioned previously, the diagnostic criteria have now been revised to a dyad of thrombocytopenia and MAHA, with no other apparent explanation for thrombocytopenia and anemia as only 10% of the patients present with the pentad, and because untreated TTP has a mortality rate (90%), it is extremely important to treat it on a presumptive basis without waiting for a definitive diagnosis [7-8].

Autoimmune diseases like SLE are one of the most common causes of acquired TTP, along with having higher mortality rates and poor response to treatment. While 71% of TTP patients have positive anti-nuclear antibodies, this could not be tested in our patients because PLEX therapy was started urgently before the test sample could be obtained. SLE-related TTP and idiopathic TTP may have overlapping clinical presentations, making it difficult to differentiate between the two. SLE patients may also present with TTP-like MAHA without a decrease in ADAMTS13 levels [5, 9].

Plasma exchange therapy and corticosteroids are the first-line treatment options for acquired TTP. The 2012 American Society of Apheresis Consensus Conference on TTP defines the response to treatment as a platelet count >150 x 103/μL for two consecutive days, a normalizing lactate dehydrogenase (LDH), and stable or improving neurologic deficits [10].

Our patient with failure of platelet response after 7 days of PLEX was diagnosed as having refractory TTP, and additional treatment options were explored.

Rituximab, an anti-CD20 monoclonal antibody used in the treatment of refractory or relapsed TTP with a once-weekly dose of 375 mg/m2 for 4 weeks being the most common regimen [1, 11]. By depleting B-cells, rituximab suppresses anti-ADAMTS13 antibody production [12]. In acute episodes of TTP, adding rituximab in the initial treatment regimen led to remission in >90% of patients within 14-21 days. Adding rituximab to PLEX and corticosteroids in the treatment regimen of patients with refractory TTP has led to increased platelet counts in >80% of patients along with decreased time for treatment response. Lim et al. suggest that rituximab be considered for initial treatment for TTP with PLEX and corticosteroids [13].

Furthermore, Eliot et al. reported in their systematic review that six out of eight studies demonstrated significantly lower relapse rates in patients receiving rituximab as compared to those receiving conventional treatment (odds ratio, OR: 0.40, 95% confidence interval, CI: 0.19-0.85). They also reported significantly higher mortality in the conventional treatment group than in the rituximab group during the follow-up (OR: 0.41, 95%CI: 0.18-0.91) [14].

Ma et al. reported that low-dose rituximab (100 mg weekly for 4 weeks) is also particularly effective in the treatment of TTP with SLE, resulting in platelet count normalization, significant reductions in B-lymphocyte counts, with TTP being in remission for 24  months of follow-up [15].

Another drug, caplacizumab was approved in February 2019 by Food and Drug Administration (FDA) for acquired TTP. It works by preventing interaction between vWF and platelets, thus blocking microthrombus formation. Various trials, particularly the TITAN and HERCULES trials showed that the drug indeed decreased mortality and morbidity of acquired TTP [12], and reduced time required for normalization of markers of end-organ damage [16]. Caplacizumab along with conventional treatment also prevents platelet aggregation more rapidly than the latter alone, thus preventing short- and long-term ischemia-related end-organ injury [17]. Scully et al. in their Phase III clinical trial comparing caplacizumab with placebo reported that the median time for platelet count normalization was shorter with caplacizumab than with placebo [2.69 days (95% CI, 1.89-2.83) vs. 2.88 days (95% CI, 2.68-3.56, p=0.01), with a statistically significant decrease (67% lower with caplacizumab) in the incidence of recurrent TTP [18]. The major side effects of the drug include epistaxis (29%), headache (21%), and gingival bleeding (16%) [18].

A debate also exists about using caplacizumab as a first-line agent for acquired TTP or reserving its use for refractory cases. Caplacizumab is currently designated an ‘orphan drug’ status and is extremely costly. Goshua et al. reported that the average cost of caplacizumab treatment in the US is $270 000 for a typical episode of TTP [19]. This might act as a deterrent to hospitals from using the drug as a first-line agent for acquired TTP from concerns for overtreatment of less severe patients, and high out-of-pocket expenditure for patients with problems in obtaining approval from insurance companies [12].

However, even after administration of caplacizumab, our patient’s platelet count failed to rise to 150 x 103/μL and plateaued around 110 x 103/μL. This might be due to bone marrow suppression due to chronic disease, i.e. SLE, as evidenced by the hypocellular bone marrow of the patient. 

Some other immune-modulatory agents might be tried in such instances like vincristine, cyclosporine, and cyclophosphamide. Other novel drugs which are currently being studied include N-acetylcysteine, which acts by reducing large vWF multimers and inhibiting vWF-dependent platelet aggregation, bortezomib, a proteasome inhibitor targeting plasma cell depletion, and recombinant ADAMTS13 [12]. Anti-CD20 antibodies, ofatumumab and obinutuzumab, can also be considered as an alternative in patients with immune TTP who were intolerant to rituximab [20].

The need for research for these drugs gains even more relevance when we consider the cost of caplacizumab, thus limiting its availability to tertiary healthcare centers [12].

## Conclusions

Thrombotic thrombocytopenic purpura is a life-threatening condition that necessitates prompt intervention. The likelihood of TTP is increased in SLE patients, and so physicians should have a low threshold of suspicion for TTP in patients presenting with thrombocytopenia and MAHA. The PLASMIC score offers a rapid and effective method to assess the likelihood of TTP and thus initiate prompt treatment. The standard treatment regimen is PLEX therapy and corticosteroids. The advent of newer drugs, mainly rituximab and caplacizumab have brought significant advancements in the management of TTP, leading to improved patient outcomes. Other drugs which can be used include immunosuppressants and anti-CD20 antibodies.
